# Portal for Families Overcoming Neurodevelopmental Disorders (PFOND): Implementation of a Software Framework for Facilitated Community Website Creation by Nontechnical Volunteers

**DOI:** 10.2196/resprot.2675

**Published:** 2013-08-06

**Authors:** Xin Cynthia Ye, Isaiah Ng, Puya Seid-Karbasi, Tuhina Imam, Cheryl E Lee, Shirley Yu Chen, Adam Herman, Balraj Sharma, Gurinder Johal, Bobby Gu, Wyeth W Wasserman

**Affiliations:** ^1^Centre for Molecular Medicine and Therapeutics, Child and Family Research InstituteDepartment of Medical GeneticsUniversity of British ColumbiaVancouver, BCCanada; ^2^PFOND Volunteer EditorVancouver, BCCanada

**Keywords:** medical informatics, medical genetics, inborn genetic disease, rare disease, social media, consumer participation

## Abstract

**Background:**

The Portal for Families Overcoming Neurodevelopmental Disorders (PFOND) provides a structured Internet interface for the sharing of information with individuals struggling with the consequences of rare developmental disorders. Large disease-impacted communities can support fundraising organizations that disseminate Web-based information through elegant websites run by professional staff. Such quality resources for families challenged by rare disorders are infrequently produced and, when available, are often dependent upon the continued efforts of a single individual.

**Objective:**

The project endeavors to create an intuitive Web-based software system that allows a volunteer with limited technical computer skills to produce a useful rare disease website in a short time period. Such a system should provide access to emerging news and research findings, facilitate community participation, present summary information about the disorder, and allow for transient management by volunteers who are likely to change periodically.

**Methods:**

The prototype portal was implemented using the WordPress software system with both existing and customized supplementary plug-in software modules. Gamification scoring features were implemented in a module, allowing editors to measure progress. The system was installed on a Linux-based computer server, accessible across the Internet through standard Web browsers.

**Results:**

A prototype PFOND system was implemented and tested. The prototype system features a structured organization with distinct partitions for background information, recent publications, and community discussions. The software design allows volunteer editors to create a themed website, implement a limited set of topic pages, and connect the software to dynamic RSS feeds providing information about recent news or advances. The prototype was assessed by a fraction of the disease sites developed (8 out of 27), including Aarskog-Scott syndrome, Aniridia, Adams-Oliver syndrome, Cat Eye syndrome, Kabuki syndrome, Leigh syndrome, Peters anomaly, and Rothmund-Thomson syndrome. The editor progress score was used to measure performance for a portion of sites.

**Conclusions:**

The PFOND system provides a convenient and structured Internet resource for the facilitated creation of information resources for families confronted by rare disorders. The system empowers volunteers to participate in the creation of quality content, while allowing for the inevitable turnover of contributors over time. The next phase of PFOND development will focus on volunteer participation in system development and community engagement.

## Introduction

The general public uses the Internet as a primary source to obtain health information, with roughly 4% of all Internet queries being health-related [1]. The majority of Internet users have obtained health information online [2,3]. There are rich sources of online medical information, exemplified by MedlinePlus, WebMD, the Mayo Clinic information portal, and Yahoo! Health [4]. Genetic disorders constitute an important subset of human disease in which DNA sequence variations fully cause or partially contribute to the disease [5]. Information about genetic diseases can be accessed online for many disorders [6-8], with the Online Mendelian Inheritance in Man (OMIM) site being one of the most enduring disease information resources [9]. Wikipedia, one of the most widely read health information resources, contains articles about many disorders [10,11]. For individual disorders, online communities may form to share information and to provide support for families struggling with the arising challenges. The popular Facebook and Twitter platforms host more than 200 breast cancer groups and 500 diabetes groups [12]. Diverse resources have been developed commercially to promote exchange between patients (eg, PatientsLikeMe) [13], while some robust communities participate in discussion boards such as that provided by Yahoo (eg, EyesApart for strabismics) [14].

A subset of genetic disorders benefits from strong support by nonprofit or academic organizations that deliver high-quality content through polished websites. For instance, the Huntington Disease Society of America maintains a website providing extensive information for families afflicted with the disease [15]. Similarly, the Cystic Fibrosis Foundation supports CF families with informative content [16]. Unfortunately, such robust information and resources are not available for many families confronted with rare genetic disorders [10], where rare refers to disorders arising in less than ~5 per 10,000 individuals [17]. While each disorder by definition impacts a small number of families, an estimated 7000 rare disorders impact a substantial population. For example, 25-30 million patients in the United States and 27-36 million patients within 25 European Union countries are impacted. When including a family member or a caregiver, the number of people directly affected by rare disorders approaches 100 million in the United States and the European Union [18]. Resources with broad coverage of rare genetic disorders include OMIM, GeneTests, and OrphaNet. These sites provide information for research professionals but have limited content for the general public [19,20]. The challenge posed to rare disease communities is enormous. It is recognized that such communities may receive less attention for the development of therapeutics [21], expert clinicians are more likely to be geographically inaccessible [18], and they may never encounter another family facing the same challenges [18,22].

Families confronted with rare genetic disorders face the challenge of finding useful information about the characteristics of the disease, guidance for the care of the afflicted family member, and news of emerging research findings [17,23,24]. Periodically a website or Facebook page may be created providing some information or links, but such resources are usually poorly maintained over time [25]. The developers of these sites may not have the technical skills to provide a complete set of features, may lack the communication skills to convey complex topics to a lay audience, and in most cases will have limited endurance for the hard work required to communicate within a small global population of interested individuals, especially if simultaneously struggling to provide care to impacted family members [26,27].

We have developed an Internet-based information portal designed to allow volunteer editors to manage delivery of information about rare disorders. Originally envisioned with a focus on neurodevelopment and subsequently broadened, the Portal for Families Overcoming Neurodevelopmental Disorders (PFOND) is a prototype Internet service that provides a basic set of automated functions and information about selected rare disorders. It delivers news items and summaries of recent scientific articles about a disorder and can support a discussion board when moderators are available. When a member of the public is available to participate in the management of a topic, the system allows for their participation in the editorial process. As a volunteer editor, the individual can compile more extensive information about a disorder, create articles, incorporate links to information resources, and oversee the discussion board. At the conclusion of a volunteer editor’s effort, the enriched content is maintained until a new volunteer is engaged to focus on the disorder page. This novel fusion of dynamic and static content within PFOND is intended to provide a basic ongoing functionality, while allowing enriched content when dedicated volunteers are available. As this report focuses on the initial development of PFOND, we assess the fraction of initial sites successfully developed and the first phase development of a gamification-based scoring procedure to encourage editor participation. In addition to providing information for and promoting the sharing of experience between families struggling with rare genetic disorders, PFOND is intended to draw clinical research and social attention to rare genetic disease as a whole.

## Methods

### Content Management Software

To facilitate the long-term maintenance of PFOND, the websites must be readily modified and moderated by individuals with limited technical expertise. Highly customized disease information pages are difficult to maintain without a dedicated administrator or Web developer. Depending on available resources, and acknowledging the dedicated and long-term efforts of some developers, such sites may stagnate with little new content and outdated styling. The use of Content Management Systems (CMS) reduces the upkeep cost by providing graphical interfaces through which volunteer editors can efficiently manage a site [28].

WordPress is an open-source blog tool and publishing platform implemented with PHP scripting language and MySQL database software. It is best known for its free hosting service, which is streamlined for blogging [29]. The software provides many additional features that make it a suitable CMS for this project [28]. It has been used to build successful sites for businesses, education, media, and others. This popularity has resulted in a broad community of users worldwide familiar with the software interface and a strong pool of online and published user guides and training manuals [28].

WordPress was chosen over other potential systems (eg, Drupal, Joomla!) for the following reasons:

- Its popularity means there are a variety of available “plugins” (software that can be installed into the system to provide additional functionality), many of which are suitable for inclusion in PFOND.
- It is maintained by hundreds of developers and contributors who provide ongoing support, additions, plugins, and fixes, thus minimizing the risk of the system becoming obsolete.
- The administrative interface (used by editors to manage content on the site) is preferred over other systems as it is easier to learn and navigate.
- As an open-source project, the software is freely available for this unfunded, community service project.

### Website Customization

To minimize development costs, WordPress plugins were used extensively to implement system functionality, including discussion board and newsfeed features. Table 1 lists the plugins that are used in the PFOND system. Additional plugin information can be found on the WordPress website [30].

Specific features not available from existing plugins had to be implemented *de novo*. By default, WordPress allows only two types of content to be created: *posts*, which hold dynamic, blog-like entries; and *pages*, which are meant to hold static content such as site or contact information. To provide support for customized sections of the site, new *post types* (types of content) and *page templates* (PHP scripts that control how a page displays information) were added. These custom features facilitate or allow editors to add and manage a list of information about a disease, research experts, and external sites related to the disease; the About, Experts, and Links pages to automatically sort and display information and provide options for visitors to navigate through them; and the News Page to properly display items generated using the automatic feed syndicator.

In addition, custom widgets allow editors to configure content in their site’s one or more sidebars. For example, widgets can show Google results for any search term, a list of new members, and recent forum posts.

**Table 1:** Utilized WordPress plugin modules.

Plugin	Author	Description
Achievements	Paul Gibbs	Allows for the gamification of certain Wordpress actions
Adminize	Frank Bültge	Fine-tune user access to backend functions by hiding unnecessary items from WordPress administration menu
BuddyPress	Open source	Provides social networking functions such as user profiles and forums
FeedWordPress	Charles Johnson	Provides Atom/RSS syndication to collect articles for the News Page
Multisite Global Search	Alicia García Holgado	Adds the ability to search the content of the individual disorder websites
Multisite User Management	Brent Shepherd	Assigns default roles for new users that join the site
Site Creation Wizard	Jon Gaulding, Ioannis Yessios	Allow users to create a site using predefined templates
Widget Context	Kaspars Dambis	Controls which widgets are displayed on which pages

### Site Theme

The typical visitor will never see the back-end system. Unless they volunteer to participate in the editorial process, users will engage the system through the graphical front-end. Hence, the website’s design plays a significant factor in attracting and serving its target community. The fonts, colors, and layouts of WordPress sites are controlled by “themes”—many of which are available for download online. For the pilot project, a customized PFOND theme was created to establish a look and feel for future PFOND sites. The following factors were taken into consideration:

- Navigation: The site should be easy to navigate, allowing new visitors to quickly find the information they seek. This has been accomplished, in part, by keeping a relatively “flat” menu hierarchy.
- Simplicity: A cleaner design is preferred to minimize loading times and allow nontechnical users to more easily navigate the site.
- Customizability: Colors, fonts, banners, and other elements can be changed without disrupting the design.

### Multisite and Site Templates

Two important aspects of PFOND are the ability to generate new disease sites in a simple manner, and the capacity to share selected forum posts, news, and disease information across sites. It is expected that a network of sites can have a greater impact on disease communities through the exchange of ideas and information. These goals were achieved using the WordPress multisite feature, which allows multiple, independent sites to operate under a common WordPress installation. Using plugins and custom scripting, the multisite feature can be adapted to include global search results and discussion boards.

The site creation process was designed with simplicity as the primary consideration. Administrators generate a new site by filling in a form to specify the desired URL, disease name, and site title. New sites are created using a default standard template, and each site allows for independent but limited customizations. The standard template contains a set of predetermined widgets, sample pages, and posts, and other visual settings, which enable editors to set up the site with few steps and therefore maintain a focus on site content.

### Additional Software Technologies and Download Information

A dedicated virtual server running CentOS was established to host the system. The following technologies were used in the development of the PFOND prototype: HTML, CSS, JavaScript, PHP, MySQL, and Apache Web Server. The software components introduced in this manuscript can be downloaded from the GitHub software repository [31].

### Motivation Mechanisms Within the Pilot Project

Two phases of testing were performed to assess system stability and usability. We recruited 8 volunteers in the alpha phase and an additional 74 volunteers in the beta phase. Two motivation mechanisms were introduced: communication and gamification-based feedback scoring. Personal email communications and small group meetings were conducted, informing volunteers about the project and encouraging their engaged interest. As the project scale increased, we implemented an achievement scoring system and other gamification features to guide the editors through the website construction and allow them to assess their performance relative to that of other editors.

## Results

### Overview of the PFOND Content

**Figure 1:**
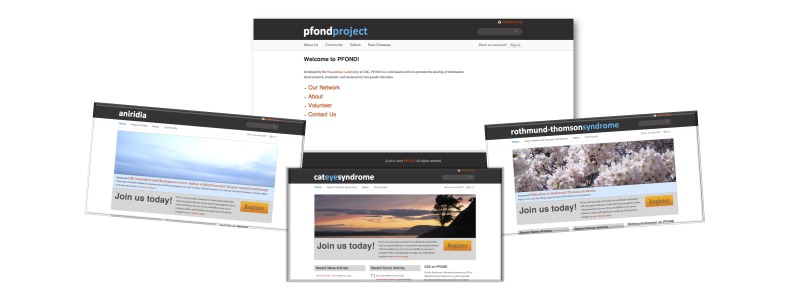
Introducing PFOND. Screenshots of the PFOND system homepage surrounded by images of selected disorder home pages, including aniridia, Cat Eye Syndrome, and Rothmund-Thomson syndrome.

**Figure 2:**
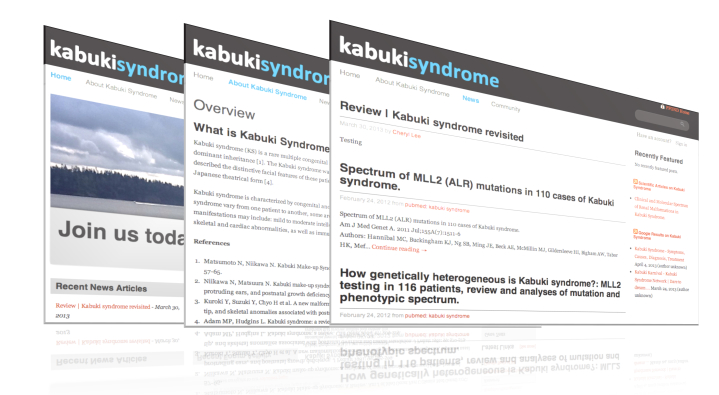
Selected components of a PFOND site for Kabuki Syndrome (Home page, About page, News page).

The PFOND system was implemented as described above in the Methods section. The resulting website will be described as a walk-through of the key pages encountered by the users of the system. For each disorder, four sections are generated, including a home page, a disease information page, a news page, and a discussion board. The pages are illustrated in Figures 1 and 2. The editor interface is described in a subsequent section.

The PFOND homepage for a disorder provides a summary of recent information. The editor can supply a front-page feature article, while the remaining sections are automatically extracted from online sources or internal pages of the system. In addition to the feature article, the page displays recent news articles about the disorder (obtained from Google News), recent internal posts from the “Community” or online resource links (from system users), and notifications about changes to the PFOND system.

There are four key subsections for each disorder. The “About” page contains information about the disorder, including a sidebar that facilitates navigation. The “Overview” section describes the characteristics of the disorder and the frequency in different populations. The “Diagnosis and Treatment” section provides information about available tests and treatments. The “Research” section provides an academic perspective of the disease, including an introduction of the underlying mechanisms when known. While the creation of the material displayed on this page represents the greatest contribution of volunteer editors, it is static and therefore the last modification date is clearly presented to inform readers of its currency.

The “Experts” page contains information about researchers active in areas related to the disease, and summaries about their work convey how the disease is currently studied. In addition to the informative summaries of research interests, a link to a website of each identified expert is provided to facilitate access to emerging findings (if available). The information on this page is particularly helpful to a new volunteer editor attempting to build upon the foundation of a departed volunteer. In the testing phase of the project, concerns were raised about providing clinical expert contact information without authorization or in-depth knowledge. As volunteer editors are not qualified to judge the quality of practitioners, the focus of this page is restricted to active researchers in the field and information presented is obtained from each researcher’s website. A warning is provided to readers to indicate that the list does not represent an endorsement or recommendation of the listed individuals.

The “News” pages present articles related to the disorder, from both lay and scientific sources. When a volunteer editor is engaged, the editor can select articles to feature on the system, providing a brief summary of the content and a link to its original source. The content of this page is predominantly produced by automated methods set-up and refined by a volunteer editor. Two key sources are displayed. A news feed is presented using the Google Alerts service, which is constrained by a set of search terms specified by the editor. Scientific articles reported in the Medline database are obtained from the PubMed RSS Feed service in a similar manner. This automation allows the user community to find recent information despite the transient attention of volunteer editors.

While editors are active, readers can use the discussion board under the “Community” page to share their understanding, questions, and stories with other members. Discussion boards provide a record of past issues for future readers to draw upon. Furthermore, the social networking on discussion boards can provide peer support for families. However, discussion boards are the most challenging aspect of PFOND. Maintaining high-quality content and minimizing “spam” and inappropriate text requires editorial review of the board contents. Given the necessity of timely posting and the focus on volunteer editors as moderators, manual review of these posts may not be possible. Blogs with large and active readership have found community-based methods to “vote down” bad content, but the nature of a rare disorders system means the readership may be strikingly small and therefore unable to support such participatory filtering. While the PFOND system has been implemented with discussion board features, the board is activated only when a volunteer indicates a commitment to facilitate the discussion on a regular basis. In the discussion section, we raise the challenging issue of how nonexpert editors should handle difficult questions or situations arising in a discussion board.

### Editor Interface

**Figure 3:**
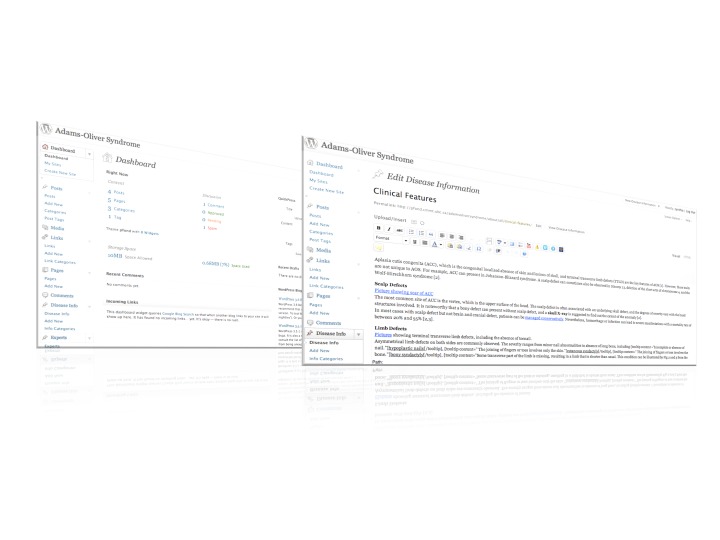
Selected views of the PFOND editor interface.

The operating interface (Dashboard) used by volunteer editors is structured in a simple format to serve two purposes (Figure 3). First, we seek to minimize the time required for volunteers to learn the system. Many of the options presented by default in the Wordpress Dashboard were removed, leaving the volunteers with less distraction. Second, the removal of or limited user access reduces the chance for unintended disruptions to the PFOND service.

Editors can modify their user name, email address, and other meta information in the Profile tab in the dashboard. In addition, they can replace the homepage picture, adjust the color scheme, and add links and RSS feeds to the site through options in the Appearance tab. The average volunteer will spend the greatest portion of his or her time editing content that appears in the Disease Info tab, rather than having to address technical matters.

### Pilot Project

To validate the software and confirm the utility of the interface for efficient volunteer utilization, a two-phase pilot project was performed.

#### Volunteer Recruitment

The model for PFOND is dependent upon the recruitment of volunteer editors to provide content and facilitate creation of new disease sites. In order to test the system, two stages of volunteer recruitment were conducted. In the first alpha-phase, volunteer requests were distributed within the University of British Columbia (UBC) Department of Medical Genetics and the Centre for Molecular Medicine and Therapeutics, seeking referrals to potential volunteers. A set of 8 individuals responded and registered on the system. An introductory in-person session was conducted. Four disease sites were initiated. In the beta phase, we recruited an additional 74 volunteers through announcements on UBC Career Services volunteer board, all physically based in the surrounding community (in order to offer an initial face-to-face introduction to the project, attended by 20 volunteers). A total of 27 disease sites were launched, of which five were topics specifically requested by volunteers.

#### Testing the PFOND Software

The testing of the system was performed by the recruited volunteer editors, following the process depicted in Multimedia Appendix 1. The goal of the initial study with 8 volunteers was twofold: to test the stability of the system and to test the usability of the editor interface. The identified system problems (10 in total were recorded) were addressed immediately by the website programmer, and the suggestions on design were compiled and discussed for potential future incorporation. In the second phase, we sought to further test the stability of the online system and gather feedback about new features. Moreover, we shifted from a combined training and communication method (online and face-to-face) to an online-only approach. The beta phase of testing was intended to allow transition from local volunteers to a global community. The prototype testing was limited to a set of basic aspects, including ease of use, customizability, community access, and automated news aggregation. For ease of use, the beta-testing process confirmed that the volunteer editors could use and maintain their sites without advanced technical knowledge. A subset of editors elected to customize fonts, colors, headers, and menus, confirming the capacity of the system to facilitate customization. Each volunteer editor established an automatic news aggregation feed by establishing and managing subscriptions to news and scientific articles. With 8 “published” PFOND sites online, the process confirmed that new disease sites could be created with relative ease.

#### Feedback Mechanisms

Multiple approaches were tested for providing feedback to volunteers about their progress. Each volunteer editor had the opportunity to file a monthly report indicating his or her work on the system, to which directed responses were provided. Such reports were used as a means for tracking the engagement of the volunteers and to identify disease sites to which additional editors could be assigned. Volunteers could request a review of their work and an assessment of the work remaining to bring the quality of their disease site content to a level suitable for release of the site to the public, which occurred for 4 out of 23 volunteer teams.

**Figure 4:**
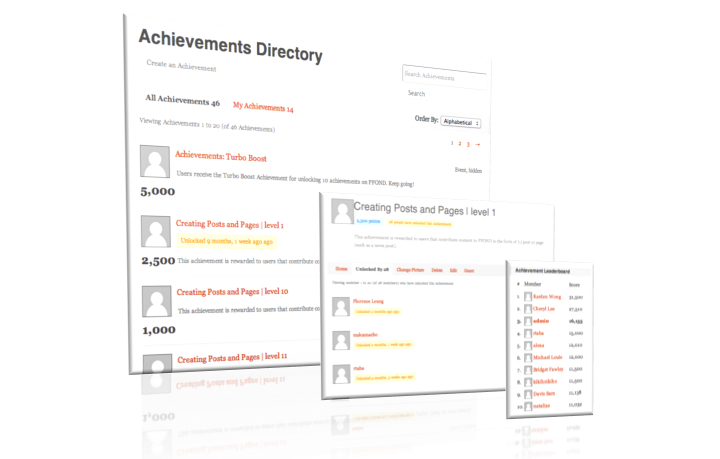
Achievements scoring system.

Based on previous experience with development of the Transcription Factor Encyclopedia [32], the developers recognized that editors benefit from measureable goals. Thus, a scoring procedure was implemented in which editors were automatically assigned points for specific tasks to guide and motivate editors throughout the development process (Figure 4). Information about some rewards was provided on the system, while other rewards were surprises intended to encourage volunteers to surpass the minimal goals specified (Table 1). The achievement system was part of a broader gamification strategy to engage participants (Table 2). Gamification is not limited to digital technology [33,34], and meaningful gamification requires user-centered design [35]. The use of external rewards to control behavior risks the nongame context being perceived negatively [35]. Adopted from Deci and Ryan’s “self-determination theory”, three innate needs for intrinsic motivation have been identified and described in the context of gamification [34,36]. Within our initial framework, PFOND meets some of these criteria. Since users can integrate the activity with their personal goals and needs, the activity is likely to be perceived as positive [35]. Table 2 lists the point values assigned to specific achievements by editors. The contributions of volunteers can be assessed in part by the points earned. Table 3 shows the coverage of gamification mechanisms within the current implementation.

#### Released PFOND Sites

A total of 8 PFOND sites have been released (ie, “published”) on the system, of which 4 were created entirely by volunteer editors and 4 were created with participation of the development team. The initial pre-alpha testing of the site focused on aniridia, for which the Aniridia Foundation International (AFI) allowed us to incorporate information from their outreach website. An automated message indicates the source and encourages readers to go to the AFI site for the most current information. The published volunteer contributions address Aarskog-Scott Syndrome (ASS), Cat Eye Syndrome, Kabuki Syndrome, and Rothmund-Thomson Syndrome. Three were assigned topics and one (ASS) was a volunteer-requested topic. The volunteers with published websites are included as authors on this report, consistent with their important contributions to the project.

**Table 2:** PFOND feedback scoring.

Entry	Score	Expected
New editor status	2500	2500
Monthly report	1000/month	3000
Image	2500/image	2500
Forum activities (PFOND-wide activities)	2500 for the 1st time, 5th time, 10th time; 1000 for the 20th, 30th, 40th, etc, up to 90th	7500
Profile update (hidden)	500 for the 1st, 3rd, 5th	1000
Tooltip activation	1000 for the 1st, 5th, 10th; 5000 for the 25th	3000
RSS feeds	2500/each	5000
News	For the 1st post: 2500; from the 2nd to the 10th post: 1000/each; 2500 for the 20th, 30th, 40th, etc	15,500
Research expert	1000/each, up to 3000 points	3000
Submission for site review	1000/each	2000
Approval for site	20,000	20,000
Disease information	1st: 2500; 5th, 10th, 25th, 50th: 1000/each; 100th: 5000	5500
Total		70,500
Maximum		100,000

**Table 3:** Relationship between PFOND and the three innate needs for successful motivation by gamification as proposed by Deci and Ryan [33,35].

Intrinsic motivation	Principle	Applications in PFOND
Relatedness	Personal goals	Editors registered in the system share a common goal: to serve the rare disorder community.
	Connect to a meaningful community of interest	The editors are connected by their common goals and interact with users with the same interest. Within each group, editors can discuss how to finish the goal together.
	The meaningful story and the social context meaning	Volunteers are aware of the needs of or have connections to the rare disorder community.
Competence	Provide interesting challenges	There are two independent challenges being a PFOND editor: one, master the system; two, create content for the website.
	Provide clear, visual, varying, and well-structured goals	See Figure 4 and Table 2.
	Provide juicy feedback	Editors get feedback from other volunteers and users.
	Beware of unintended behaviors	The points are tuned to specific actions aligned with the editor motivation, decreasing the likelihood of alternatives.
Autonomy	Play is voluntary	All editors in PFOND are volunteers, and there is no requirement to monitor the scores provided.
	Beware of losing autonomy	We focus on individual accomplishment. The structure encourages tasks to be completed, allowing the editors to shape the content at each step in the manner that they determine.
	Beware of devaluating activity	Working on rare disorders gives a strong value to the activities of the editor. We build on this key motivation in each step of the project, from recruitment to completion of a functional site.

## Discussion

### Principal Findings

The PFOND project provides an intuitive Web-based software system to enable the creation and maintenance of websites for rare genetic diseases. It is intended to allow nontechnical editors to participate in the creation and delivery of disease information informed by scientific research. Grouping services for diverse disorders within one framework offers several potential advantages: (1) greater public awareness of available resources, (2) shared technical challenges to allow volunteers to focus on quality content rather than system management, (3) the capability to share information across communities where appropriate, and (4) the opportunity for transitioning between volunteer editors over the long-term to accommodate the transient nature of volunteer editor service. Most importantly, PFOND aims to provide a cyber home for patients and their families.

The development of PFOND addresses an information gap for rare diseases. Key resources can be identified for each disorder and presented in an easily accessible format. Although the information in no way diminishes the necessity for professional advice and counselling, families provided with improved online information about emerging clinical research may develop a deeper understanding and therefore be better equipped to discuss matters and concerns with their health care providers [37-39]. Ideally, the information presented would accelerate the dissemination of information about identification of beneficial approaches to the disorder, such as available treatments, dietary changes, or useful devices [39,40].

Online resources such as PFOND promote awareness that there are other families facing similar challenges, which may help alleviate any sense of isolation [18,24]. For sites with an actively moderated discussion forum, users will have the opportunity to directly engage with peers to gain insights from their experience and diverse information sources [41,42]. While there are risks of inaccurate information, some studies suggest such problems may be infrequent [43,44]. Nevertheless, the risk remains that users may present claims and suggestions that may be inaccurate or inappropriate. While moderation can reduce the potential problem, the fact that PFOND is maintained by volunteers with no requisite training results in two choices: (1) exclude the benefits of forum interactions from the site or (2) include a clear warning to users that information presented in forums may not be reliable. Recent online services may help alleviate the challenges. Broader access to qualified medical professionals, such as provided by HealthTAP [45], may alleviate the tendency of users to seek medical advice from forums [45,46].

The online community is dynamic and growing. Increasingly individuals are turning to the Internet for volunteering and opportunities to participate collectively in projects with positive social impacts. The most novel aspect of PFOND is the focus on the volunteer for the creation and dynamic development of websites focused on communities impacted by rare disorders. An enduring volunteer-powered resource allows for an online community to form and to continually benefit from new information and insights into the challenges faced at different life stages by those impacted by a disorder.

### Future Directions

Three areas of activity are expected in the next iteration of PFOND development. First, the existing system can be extended to better support users with accessibility challenges, such as limited vision. Second, the system can be made available to a broader range of volunteer editors who can contribute to building and improving the site. Third, the framework of PFOND will allow for structured, quantitative research of both user and volunteer editor engagement/satisfaction and editor performance when provided with distinct gamification features or innovative design elements. In the longer term, there may be opportunities to promote exchange between related PFOND communities. Each of the future directions brings challenges.

Users of PFOND are more likely to be challenged by impairments than the average Internet user. Thus, the further development of PFOND will require greater attention to the issue of website accessibility, which can be addressed by increasing the flexibility of the design. The pilot version has been designed to be easily navigated, but additional work will be required to make it accessible, particularly for users with low vision [47]. While the use of WordPress as the underlying system will allow assistive software to be applied [48], there are additional steps that can be taken in the next iteration of PFOND. Users can be empowered to change display settings to use larger font sizes, increase line spacing, and apply more contrasting colors. Site editors will need structured training to configure these options to suit their target audience (for example, the aniridia site could use more readable fonts by default). The introduction of the assistive features is a key step moving forward.

The ultimate goal for PFOND is the engaged participation of volunteer editors from around the globe. The current prototype was tested within the geographic confines of one university, with extensive oversight of the initial cadre of participants. In the second phase, the system can be modified to minimize supervisory time, allowing senior volunteer editors to take on additional supervisory activities, while empowering the development team to maintain an overview of the material being posted each day. In the second phase, we will disseminate calls for participation across volunteer recruitment boards, both in global posting boards (eg, VolunteerMatch) and in directed fashion to volunteer boards serving specific communities such as university students [49].

Appropriate gamification mechanisms are especially important for the system to succeed on a large scale. Inappropriately implemented gamification could decrease editor engagement, creating a perception of decreased social value if such extrinsic motivation mechanisms are required [35]. An irrelevant and enforced scoring system relying on points, leaderboards, and badges can do more harm than good. The current PFOND system has emphasized intrinsic motivation (as seen in Table 3), in addition to points and leaderboards. When users are able to connect their goals to values they hold, a better outcome might be expected. However, there is limited published research on the effectiveness of game-like elements to promote patterns of activity within Internet-based community service systems. We will implement additional gamification features, including badges, and measure how effective they are in motivating editors to finish a website, using the PFOND framework to compare and contrast distinct approaches on the successful completion of PFOND sites and on user satisfaction and perception.

For such measurements, one potential user survey is WAMMI (Website Analysis and Measurement Inventory), a 60-item questionnaire, but an average user may be unlikely to complete such a long instrument [50]. A shorter survey developed by van Schaik and Ling (2009) assesses user perceptions of ease of use, utility disorientation, flow, and aesthetics [51]. The reliability and validity of the scales have been confirmed. The experience of editors can be measured with a different survey, as they use a different interface with less Web-like design. The Software Usability Measurement Inventory (SUMI) is intended for such non-Web user interfaces [52] but conveniently shares similarities with WAMMI [51].

The PFOND site incorporates some innovative design features, but in the next phase it will be possible to measure the impact of a series of features. The first phase emphasis on content building limited, but did not preclude, innovation. For instance, the PFOND site includes dynamically updating elements and static summaries in partnership to better address the transient nature of editor participation. In the next wave of the project, flexibility for innovation will be added to the system. In addition to the accessibility features addressed above, we will encourage volunteer editors to review the IDEO Methods Cards App in order to inspire potential features [53]. Through this community participation, we will identify a subset of innovative features to test within the system, providing a subset of editors with the design feature and measuring their perception using validated surveys.

The solid computational foundation introduced in this report lays the groundwork for a broader international volunteer effort for creating online resources for families struggling with rare disorders.

## Multimedia Appendix 1

Steps to establish a new PFOND site. (a) In the first step, volunteers submit an application to serve as an editor. (b) Accepted editors use an intuitive admin interface to create a PFOND site. (c) Editors collect and update their site with disease information, relevant news items, pertinent links, and field experts. (d) When ready, editors publish a PFOND site making it accessible to all readers. (e) The new site is added to the PFOND homepage and is indexed by search engines for increased exposure.

## Abbreviations

AFI: Aniridia Foundation International
ASS: Aarskog-Scott Syndrome
CF: cystic fibrosis
CMS: Content Management System
OMIM: Online Mendelian Inheritance in Man
PFOND: Portal for Families Overcoming Neurodevelopmental Disorders
SUMI: Software Usability Measurement Inventory
WAMMI: Web Site Analysis and Measurement Inventory
