# Supplementary figures and images for: Portal for Families Overcoming Neurodevelopmental Disorders (PFOND): Implementation of a Software Framework for Facilitated Community Website Creation by Nontechnical Volunteers
Source: JMIR Res Protoc. 2013 Aug 6;2(2):e25. doi: 10.2196/resprot.2675 (PMC3742411; doi:10.2196/resprot.2675)

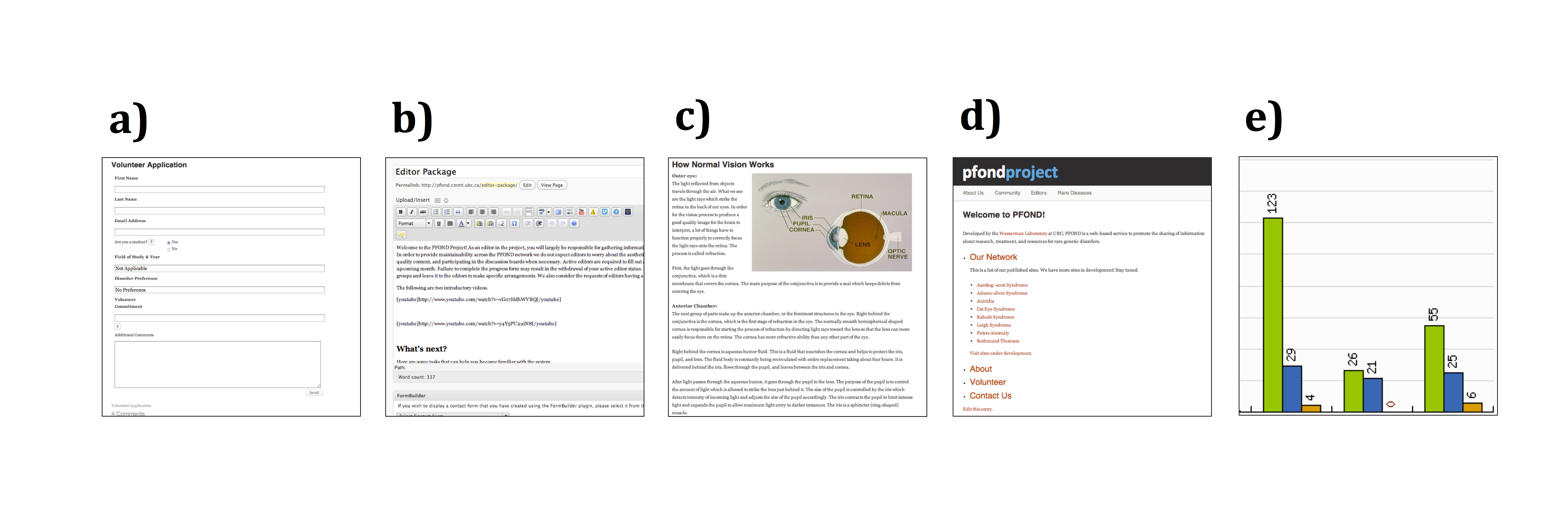

Supplement: Supplementary file 1 [file resprot_v2i2e25_app1.png]
